# Spatial-Temporal Characteristics and Influence Factors of Carbon Emission from Livestock Industry in China

**DOI:** 10.3390/ijerph192214837

**Published:** 2022-11-11

**Authors:** Dequan Hao, Rui Wang, Chaojie Gao, Xinyan Song, Wenxin Liu, Guangyin Hu

**Affiliations:** College of Economics and Management, Northwest A&F University, Yangling, Xianyang 712100, China

**Keywords:** livestock industry, carbon emissions, temporal variation, spatial feature, influence factors, China

## Abstract

Animal husbandry is an important source of carbon emissions. As a large country, China must measure the carbon emissions from animal husbandry to reveal the spatial and temporal characteristics and determine the influencing factors to realize low-carbon animal husbandry and carbon emission reduction. In this paper, the carbon emissions of the livestock industry in each province of China were calculated with the emission coefficient method, considering the temperature change factor. The spatial and temporal characteristics and influencing factors of livestock industry carbon emissions were analyzed using the kernel density model, the spatial autocorrelation model, and the Tobit model. The results indicated that: (1) From 2000 to 2020, carbon emissions from the livestock industry in China experienced four stages: rapid rise, rapid decline, slow rise, and fluctuating decline, with an overall downward trend. Carbon emissions in the eastern and central regions showed a downward trend, while carbon emissions in the western regions showed an upward trend. (2) In terms of time, the relative gap in carbon emissions among the provinces narrowed first and then widened; the spatial agglomeration of carbon emissions from livestock farming in China increased, gradually forming the characteristics of “high agglomeration, low agglomeration”, and showing a gradually decreasing pattern from northwest to southeast. (3) Nationwide, industrial structure, population, and farmers’ income levels have had significantly promoting effects on animal husbandry carbon emissions, and the urbanization and agricultural mechanization levels have had significant inhibitory effects on carbon emissions. Finally, based on the above factors, it can be concluded that recognizing the location conditions, promoting the upgrading of industrial structures, and adopting differentiated strategies will help to promote the reduction in carbon emissions in animal husbandry and achieve its high-quality development.

## 1. Introduction

Carbon dioxide emissions are considered to be the main cause of global warming [1]. The increase in agricultural carbon emissions will lead to a series of problems such as the deterioration of the agricultural production environment, the frequent occurrence of extreme weather, and the decline of agricultural output. Therefore, agricultural carbon emission reduction plays an important role in ensuring food security and maintaining sustainable agricultural development. The greenhouse gas emissions from agricultural production activities in China account for about 20% of the total greenhouse gas emissions, and the carbon emissions account for about 13% of the total national carbon emissions [2,3]. Among them, carbon emissions generated by animal husbandry in the process of livestock breeding accounted for more than two-thirds of agricultural carbon emissions. According to statistics, carbon emissions from animal husbandry increased from 335.4835 million tons in 2008 to 346.7002 million tons in 2017, with an average annual growth rate of 1.63% [4]. On the one hand, China is a large country with a population of 1.41 billion. With the increasing living standards of residents, their dietary structure has undergone significant changes, and the demand for livestock products such as meat, milk, and eggs are constantly increasing. Ensuring a stable increase in the output of livestock products will inevitably lead to an increase in carbon emissions from animal husbandry. On the other hand, the economic return of animal husbandry is higher than that of the planting industry, so more and more farmers practice animal husbandry as their way to increase income and promote its rapid development. However, the excessive increase in carbon emissions from animal husbandry will not only threaten ecological security but also hinder the realization of the “double carbon” target. Therefore, in order to meet the dietary needs of residents and increase the income of farmers, it is necessary to reduce the carbon emissions from animal husbandry as much as possible, so as to realize the “double carbon” goal. It is necessary to study and analyze the spatial and temporal characteristics and influence factors of carbon emissions in China.

Through a literature review, we discovered that existing studies have mainly focused on agricultural carbon emissions, while few studies have involved carbon emissions from animal husbandry. The research results on agricultural carbon emissions can be roughly summarized into the following three categories: first, the measurement and estimation of agricultural carbon emissions. At home and abroad, the life-cycle method, the carbon emission coefficient method, and the model method are the main ways to measure agricultural carbon emissions. Rehman A. et al. (2018) used the carbon emission coefficient method to study the agricultural GHG emissions in Pakistan and found that the GHG emissions generated by livestock farming accounted for more than half of the total agricultural GHG emissions [5]. Xie T. (2020) calculated the carbon emissions from animal husbandry in three provinces in Central China using the carbon emission coefficient method, and large livestock such as cattle and sheep were the main emission sources in animal husbandry [6]. Referring to the IPCC greenhouse gas emission inventory, Yusen L. et al. (2017) used the carbon emission coefficient method to calculate China’s agricultural carbon emissions from 1997 to 2014, and the results indicated that the carbon emissions generated by livestock farming were much higher than those generated by farming [7]. West and Marland (2002) constructed a measurement index system of agricultural carbon emissions based on the four dimensions of chemical fertilizers, pesticides, agricultural irrigation, and seed cultivation, and used the whole life-cycle method to calculate the carbon emissions in the United States as an example [8]. Yao C.S. (2017) used the whole life-cycle method to measure the carbon emissions from animal husbandry in 31 provinces and autonomous regions in mainland China from 2000 to 2014, and the results showed that intestinal fermentation and manure management accounted for about 80% of the total carbon emissions from animal husbandry [9]. Some scholars have adopted model methods. For example, Neufeldt et al. (2006) measured agricultural carbon emissions in southwest Germany based on the EFEM model and the DNDC model [10]. However, there are few existing studies on the carbon emission measurement of animal husbandry and many of them refer to previous studies by adopting a unified carbon emission factor, which ignores the impact of temperature change on the animal husbandry carbon emissions, resulting in certain errors and inaccurate results. Second, the influencing factors of agricultural carbon emissions were investigated. ACIL Tasman Pty Ltd. (2009) measured the agricultural carbon emissions of the United States, Canada, India, and other countries and found that the differences in the agricultural industrial structure and production efficiency of each country affected their agricultural carbon emissions to a certain extent [11]. Ipek Tunc G. et al. (2009) used the average Deeley index decomposition model to explore the influencing factors of agricultural carbon emissions, and the results indicated that economic activities positively promoted carbon emissions, while structural effects negatively inhibited carbon emissions [12]. Alamdarlo H.N. et al. (2016) used EKC theory to evaluate the correlation between agricultural carbon emissions and per capita GDP in Iran, and the results showed that in the early stage, the increase in per capita GDP promoted the increase in agricultural carbon emissions, but after the carbon emissions rose to a certain height, they declined with the increase in per capita income [13]. Zhao X.C. et al. (2018) used the LMDI model to decompose the influencing factors of agricultural carbon emissions in Hunan Province, China and found that the agricultural economic level and industrial structure positively promoted agricultural carbon emissions, while the agricultural production efficiency and labor force scale significantly inhibited agricultural carbon emissions [14]. Zhou Y.F. et al. (2022) used the spatial panel model to explore the influencing factors of agricultural carbon emissions in Hebei Province, China and found that the level of agricultural economic development was the main factor driving the growth of agricultural carbon emissions, while the urbanization rate had a restraining effect on the growth of agricultural carbon emissions [15]. However, existing research mostly uses the LMDI model to decompose carbon emissions, ignoring the restricted dependent variable of carbon emissions greater than 0, which may cause certain deviations in the estimation results. Third, research on the spatiotemporal characteristics of agricultural carbon emissions. Qiu Z.J. et al. (2021) analyzed the temporal changes in agricultural carbon emissions in Jiangsu Province by calculating its agricultural carbon emissions and using the STIRPAT model. The study found that the temporal characteristics of agricultural carbon emissions in Jiangsu Province from 2000 to 2019 were “down-up-down”. Based on the re-calculation of China’s agricultural carbon emissions from 2005 to 2019 [16]. Tian Y. et al. (2022) used kernel density estimation and the spatial autocorrelation model to analyze and conclude that China’s agricultural carbon emissions showed a time feature of annual decline but inter-annual fluctuations, and the overall spatial feature of carbon emission intensity was “high in the east and low in the west” [17]. Wu Y.G. et al. (2019) selected the data from 1997 to 2015 to calculate China’s agricultural carbon emissions and analyzed its characteristics from the two dimensions of space and time, suggesting that the total carbon emissions showed the three characteristics of “fluctuating rise—rapid rise—slow decline”, and the spatial distribution was obviously unbalanced [18]. However, existing studies fail to reveal the dynamic evolution trend of carbon emissions from livestock farming in China and ignore its spatial pattern and the spatial correlation among provinces, so the research conclusions are not adequate in explaining the dynamic change in carbon emissions from livestock farming.

**Figure 1:**
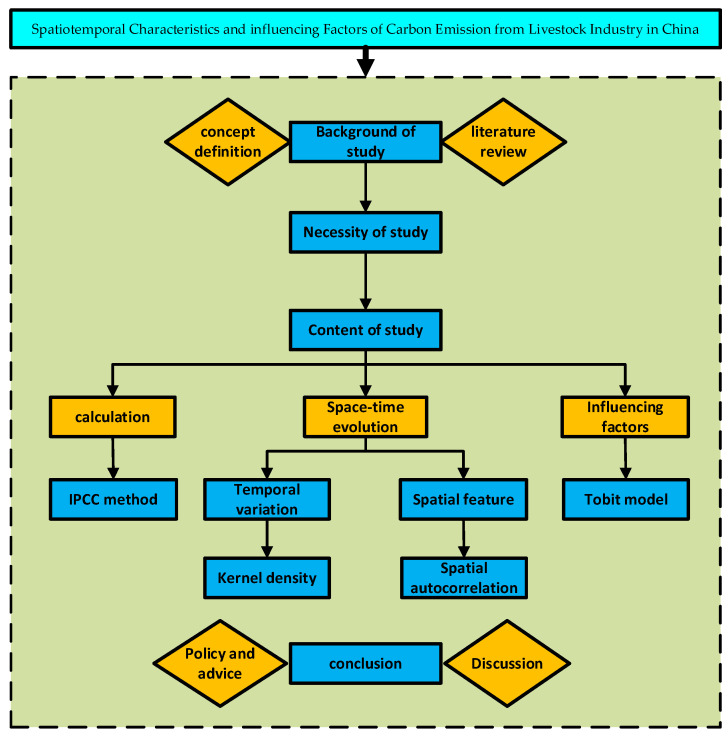
Technology roadmap.

Based on the lack of existing research, the marginal contribution of this paper is as follows: on the one hand, the carbon emissions from animal husbandry were calculated by referring to the carbon emission factors of animal husbandry determined by *IPCC 2006* [19], combined with the temperature changes over the years, making the calculation results more precise. Second, kernel density estimation was used to analyze its dynamic evolution process. The 3D kernel density method was used to reveal the carbon emissions of the livestock industry in all years, overcoming the limitation of the 2D kernel density map, which could only show some years, and clarified the time variation characteristics of the livestock industry. Finally, specific changes in a region affect not only the region itself, but also the nearby population, policies, resource allocation, and investment channels. Therefore, ignoring spatial relationships is likely to lead to ineffective decisions. Quantifying the key drivers of livestock carbon emissions by understanding the variation in time and space is the third marginal contribution of this paper. To sum up, as shown in Figure 1, firstly, we measured the carbon emissions of animal husbandry in each province in China. Secondly, by using 3D kernel density method and spatial autocorrelation model, we reveal the spatial-temporal evolution characteristics of carbon emissions of animal husbandry in China. Finally, we explored the influencing factors of carbon emission of animal husbandry in China. We expected to help to promote the reduction in carbon emissions in animal husbandry and achieve its high-quality development.

## 2. Materials and Methods

### 2.1. Carbon Emission Calculation of Animal Husbandry

The research scope of this paper was the state of animal husbandry in 31 provincial–Level administrative units (excluding Hong Kong, Macao, and Taiwan) in mainland China from 2000 to 2020. The targets were cows, non-cows, goats, sheep, horses, camels, donkeys or mules, poultry, and pigs. We took the animal husbandry-related data of 31 provincial administrative units from 2000 to 2020 as the sample. The sampling method was obtained by consulting statistical yearbooks. The reason for choosing these animals was that they were referenced in the existing literature on the calculation of animal husbandry carbon emissions.

Livestock greenhouse gas emissions comprise two parts; the first part is methane $\left(CH_{4}\right)$ from livestock in the process of intestinal fermentation, and the second part is methane $\left(CH_{4}\right)$ and nitrous oxide $\left(N_{2}O\right)$ produced in the process of animal waste management. According to the emission coefficients of different kinds of livestock in *IPCC 2006* [19], the emission coefficient method was used to calculate the relevant greenhouse gas emissions. In particular, in the calculation of methane $\left(CH_{4}\right)$ emissions from animal waste management, taking the influence of different average temperatures on the discharge coefficient into account, and determining the discharge coefficients of different provinces according to the different temperatures will result in a more accurate amount of greenhouse gas emissions (detailed in Appendix A, Table A1, limited space, only per the year, unified for °C temperature unit).

#### 2.1.1. The Average Annual Size of Livestock

Because the seasonal birth or slaughter of livestock may cause livestock numbers to increase or decrease during a year, adjustments to the average annual livestock size were required. The adjustment formula is as follows, according to *IPCC 2006* [19]:

(1) $$APP=\{\begin{array}{c} Herds_{end}\;Days_{live}\geq 365\;\\ Days_{live}\;\cdot \left(\frac{NAPA}{365}\right)\;Days_{live}<365 \end{array}$$

In Equation (1), APP is the annual average size of livestock; $Herds_{end}$ is the year-end stock of livestock; $Days_{live}$ is the normal feeding cycle of livestock (days); NAPA is the year-end quantity of livestock.

#### 2.1.2. Animal Intestinal Fermentation $CH_{4}$ Calculation

The total amount of methane $\left(CH_{4}\right)$ equals the emission coefficient of methane $\left(CH_{4}\right)$ produced by certain kinds of animals in the process of intestinal fermentation multiplied by the annual average number of the corresponding types of animals (Table 1) [19].

(2) $$E_{CH_{4,enteric}}=\sum \delta_{i}\cdot {AP}_{i}\;$$

In Equation (2), $E_{CH_{4,enteric}}$ is the total methane $\left(CH_{4}\right)$ emissions produced in the process of livestock intestinal fermentation; $\delta_{i}$ is the methane $\left(CH_{4}\right)$ emission coefficient by animal kind i during its intestinal fermentation; $AP_{i}$ is the annual average number of the kind i.

#### 2.1.3. Animal Waste Management $CH_{4}$ Calculation

The total amount of methane $\left(CH_{4}\right)$ equals the emission coefficient of methane $\left(CH_{4}\right)$ produced by certain kinds of animals in the process of waste management multiplied by the annual average number of the corresponding types of animals (Table 2, Table 3 and Table 4) [19].

(3) $$E_{CH_{4,manager}}=\sum \gamma_{i}\cdot AP_{i}\;$$

In Equation (3), $E_{CH_{4,manager}}$ is the total amount of methane $\left(CH_{4}\right)$ emissions in the process of livestock waste management, $\gamma_{i}$ is the methane $\left(CH_{4}\right)$ emission coefficient in the process of waste management of the livestock kind 𝑖, and $AP_{i}$ is the annual average number of the kind 𝑖.

#### 2.1.4. Animal Waste Management $N_{2}O$ Calculation

The total amount of nitrous oxide $\left(N_{2}O\right)$ equals to the emission coefficient of nitrous oxide $\left(N_{2}O\right)$ produced by certain kinds of animals in the process of waste management multiplied by the annual average number of the corresponding types of animals (Table 5) [20].

(4) $$E_{N_{2}O,manager}=\sum \beta_{i}\cdot AP_{i}\;$$

In Equation (4), $E_{N_{2}O,manager}$ is the total amount of emissions of nitrous oxide $\left(N_{2}O\right)$ emissions produced in the process of livestock intestinal fermentation; $\beta_{i}$ is the nitrous oxide $\left(N_{2}O\right)$ emission coefficient of animal kind i during its intestinal fermentation; $AP_{i}$ is the annual average number of the kind 𝑖.

#### 2.1.5. Calculation of Carbon Emission of Animal Husbandry

By referring to the *IPCC 2006*, this paper set the global warming potential values of $CO_{2}$, $CH_{4}$ and $N_{2}O$ to 1, 21, and 310, respectively, according to which we converted the $CH_{4}$ and $N_{2}O$ produced during livestock intestinal fermentation and waste management into $CO_{2}$. Therefore, the carbon emissions are calculated as Equation (5):

(5) $$T_{CO_{2}}=\left[\left(E_{CH_{4,enteric}}+E_{CH_{4,manager}}\right)\cdot 21+E_{N_{2}O,manager}\cdot 310\right]/10^{8}$$

In Equation (5), $T_{CO_{2}}$ is the carbon emissions from animal husbandry.

In Equations (2)–(4), their units are kg. In order to conveniently add and further analyze them, the units were converted from kg to $10^{8}\;t$ by Equation (5). Therefore, the units of carbon emissions are $10^{8}\;t$.

### 2.2. Kernel Density Estimation

In order to clarify the distribution dynamics and evolution law of carbon emissions from the livestock industry in China and its regions, the kernel density estimation method was used to analyze the distribution location, distribution trend, polarization trend, and distribution extension. Among them, the distribution position reflects the carbon emission level of animal husbandry. The distribution trend reflects the spatial difference and polarization trend of carbon emissions in animal husbandry, in which the width and height of wave peaks reflect the difference, and the number of wave peaks depicts the polarization trend [21]. The extensibility of distribution reflects the spatial difference between the area with the highest livestock industry carbon emissions and other parts of the study area, and the longer the tail, the greater the difference [22].

For independent and uniformly distributed sample data $\;x_{1},\;x_{2},\ldots ,x_{n},$ kernel density estimation is in the form of:

(6) $$\hat{f_{h}}\left(x\right)=\frac{1}{nh}\overset{n}{\underset{i=1}{\sum }}K(\frac{x_{i}-\bar{x}}{h})$$

In Equation (6), $\hat{f_{h}}\left(x\right)$ is density function; $K\left(\frac{x-x_{i}}{h}\right)$ is kernel function, h is bandwidth, n is the number of observations (the total number of provinces), i is the provinces, $x_{i}$ is independent and identically distributed observations, and $\bar{x}$ is the average.

### 2.3. Spatial Autocorrelation

According to Tobler’s first law of geography, there is a connection between things and the surrounding environment. The closer the distance, the closer the connection [23]. Spatial autocorrelation can accurately represent adjacent impacts. In other words, the geographical location of a region will not only affect its own livestock carbon emissions but will also affect the surrounding areas. The importance of spatial autocorrelation is that it helps to determine the extent to which location affects carbon emissions from livestock farming and whether there is a clear relationship between carbon emissions from livestock farming and location. China is a country with a vast territory and different resource endowments. Therefore, it is necessary to measure the spatial autocorrelation of carbon emissions from animal husbandry in China. Spatial autocorrelation is an index to measure the degree of spatial agglomeration of an attribute from the overall perspective and is mainly divided into two methods: global spatial autocorrelation and local spatial autocorrelation. 

Global spatial autocorrelation is used to determine whether a certain attribute, on the whole, has spatial agglomeration and dispersion characteristics, usually by adopting the Global Moran’s I index, according to the formula as follows [24]:

(7) $$Moran’s\;I=\frac{\sum_{i=1}^{n}\sum_{j=1}^{n}W_{ij}\left(x_{i}-x^{\prime }\right)}{S^{2}\sum_{i=1}^{n}\sum_{j=1}^{n}W_{ij}}$$

(8) $$S^{2}=\frac{\sum_{i=1}^{n}{\left(x_{i}-x^{\prime }\right)}^{2}}{n}$$

In Equation (7), n is the sample size; $x_{i}$ and $x_{j}$ are observations of property $x_{i}$ on spatial units i and j; $x^{\prime }$ is the mean value of an attribute in each spatial unit; $W_{ij}$ is the spatial weight matrix; Moran’s I index values range [−1,1]. A positive value indicates that the areas with higher (or lower) carbon emissions from animal husbandry are spatially significantly concentrated; negative values indicate that there are significant spatial differences in areas with higher (or lower) carbon emissions from livestock farming; 0 indicates that carbon emissions from animal husbandry are randomly distributed in space [25,26].

Local spatial autocorrelation is used to judge whether an attribute of each space has spatial correlation locally. The Local Moran’s I index is as follows [27,28]:

(9) $$Moran’s\;I=\frac{\left(x_{i}-x^{\prime }\right)\sum_{j=1}W_{ij}\left(x_{j}-x^{\prime }\right)}{S^{2}}$$

A positive Local Moran’s I index means a positive local correlation (adjacent regions have nearly the same kind of attribute values), a negative index means as negative correlation (different types of attribute values for adjacent areas), and the higher the degree of absolute value, the greater the adjacency [29].

### 2.4. Tobit Model

This paper calculated that the animal husbandry carbon emissions of 31 Chinese provinces (municipalities directly under the central government, autonomous regions) were greater than zero and belonged to the “limited dependent variable”. The Tobit model is a good way to solve the problem of “limited dependent variable” regression [30], so this article set up a random effects panel Tobit model to perform an empirical analysis of the influence factors of carbon emissions from 31 Chinese provinces’ animal husbandry. Based on the existing research conclusions and combined with the reality of carbon emissions from China’s livestock industry, the factors affecting the carbon emission level of China’s livestock industry included industrial structure, urbanization level, agricultural economic development level, agricultural mechanization level, the carbon production efficiency of the livestock industry, total population, dietary structure, and farmers’ income levels. To clarify the mechanism and degree of the different influencing factors on the carbon emissions of China’s livestock industry, we proposed targeted effective measures to promote a reduction in carbon emissions. In the empirical test, the carbon emissions from animal husbandry in 31 provinces (municipalities directly under the central government and autonomous regions) in China were selected as explained variables, and the following influencing factors are selected as explanatory variables (Table 6). The Tobit model formula is as follows:

(10) $$Y_{it}=\beta_{0}+\beta_{1}str_{it}+\beta_{2}urb_{it}+\beta_{3}eco_{it}+\beta_{4}mec_{it}++\beta_{5}eff_{it}+\beta_{6}lnpop_{it}+\beta_{7}lnfod_{it}+\beta_{8}lninc_{it}+\varepsilon_{it}$$

In Equation (10), $Y_{it}$ is carbon emissions for the livestock industry; i is areas; t is time; $\beta_{0}$ is constant; *str* is industrial structure; *urb* is urbanization level; *eco* is agricultural economic development level; *mec* is agricultural mechanization level; eff is animal husbandry production efficiency; pop is total population; fod is prandial structure; *inc* is farmers’ income level; $\varepsilon_{it}$ is random perturbation terms. In order to eliminate the influence of heteroscedasticity, the data of the three absolute indicators of the total population, dietary structure, and farmers’ income levels were logarithmically processed [31].

Based on the existing research conclusions [32,33,34], this study devised an expected relationship of variables (Table 7).

### 2.5. Data Declaration

The livestock production of 31 provinces from 2000 to 2020 was taken from the *China Animal Husbandry and Veterinary Yearbook*. The animal husbandry fishery output, animal husbandry output value, urban resident population, population, size of the agricultural labor force, agricultural machinery total power, crop planting area, rural resident family meat egg milk consumption per capita, and per capita disposable income of rural households and other data were taken from the *China Statistical Yearbook, China Agricultural Statistics Yearbook*, and *Chinese Rural Statistical Yearbook*. Some missing data were supplemented by interpolation. Among them, the total output value of agriculture, forestry, animal husbandry, and fishery and the total output value of animal husbandry for each year were reduced based on the year 2000 to eliminate the influence of price factors [35,36,37,38] (https://data.cnki.net/, accessed on 30 April 2022).

## 3. Result and Analysis

### 3.1. Temporal Characteristics of Carbon Emissions from Animal Husbandry

#### 3.1.1. Analysis of Carbon Emissions from Animal Husbandry

**Figure 2:**
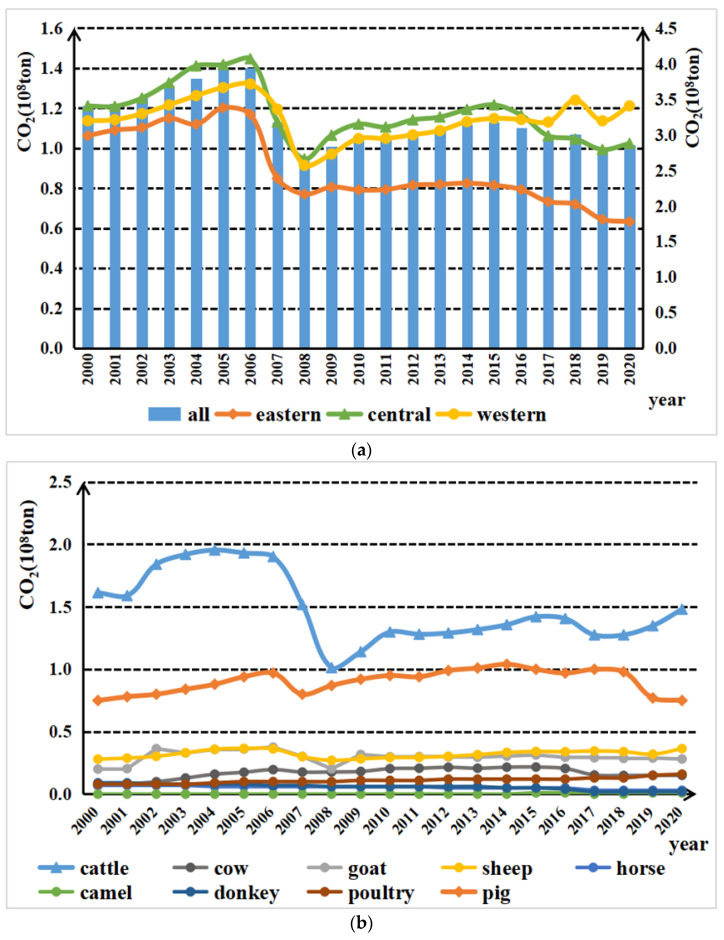
(**a**) Carbon Emissions of livestock industry in different regions, 2000–2020 (10^8^ ton); (**b**) Carbon Emissions of livestock industry in different kinds, 2000–2020 (10^8^ ton).

As shown in Figure 2a, the 2000–2020 national livestock emissions, reduced from 340.8509 million *t*$CO_{2}$-eq to 286.596 million *t*$CO_{2}$-eq, experienced four stages:

(1) The rapid rise stage (2000–2005): animal husbandry carbon emissions rose from 340.8509 million *t*$CO_{2}$-eq to 392.1207 million *t*$CO_{2}$-eq with a growth rate as high as 15.04%. During this period, influenced by the continuous increase in grain production in the middle and late 1990s, grain supply was in large excess, and the increase in farmers’ income slowed down [9]. In this context, China significantly accelerated the pace of agricultural structure adjustment, on the one hand, encouraging farmers to engage in animal husbandry production and actively promoting the scale, standardization, and industrialization, and on the other hand, adjusting the tax policies related to animal husbandry, gradually reducing the livestock tax year by year, the animal slaughter tax, and the tax rate, thus promoting the rapid development of animal husbandry. This in turn led to a sharp rise in livestock emissions, which peaked in 2006.

(2) The rapid decline stage (2006–2007): in two years or so, China’s animal husbandry carbon emissions fell from 393.5556 million *t*$CO_{2}$-eq to 317.3168 million *t*$CO_{2}$-eq with a decline of 19.37%. The possible reasons for the decline were that since early 2007, the international grain export prices rose sharply, and oil prices and transportation costs increased at the same time, facilitating the worldwide food crisis. China’s domestic grain market supply was tight, prices rose sharply, and to alleviate the pressure on the food supply, country began to restrict the grain-consuming animal husbandry. The number of non-cows, sheep, and other livestock was significantly reduced, and GHG emissions from livestock intestinal fermentation and manure management were significantly reduced, which in turn led to a rapid decline in total carbon emissions from animal husbandry during this stage.

(3) The slowly rising stage (2008–2015): the stage of animal husbandry carbon emissions rose from 262.886 million *t*$CO_{2}$-eq to 317.8069 million *t*$CO_{2}$-eq with an average annual growth rate of 2.61%. The possible reasons were as follows: with the increasing improvement in residents’ living standards, the demand for meat, milk, eggs, and other animal products continued to rise, and animal husbandry gradually recovered growth. However, compared with the first stage, because of the diversified development of the rural economy and industry, farmers’ income channels had continuously expanded, which reduced the proportion of agricultural income in the total household income to a certain extent and weakened farmers’ dependence on animal husbandry to increase income.

(4) Fluctuating decline stage (2016–2020): livestock emissions fell from 310.0198 million *t*$CO_{2}$-eq in 2016 to 286.596 million *t*$CO_{2}$-eq in 2020 with a decline of 7.65%. Possible reasons could be that on the one hand, from 2016 to 2020, China developed a series of animal husbandry carbon emission reduction policies and suggestions, such as *the Ministry of Agriculture and Rural Affairs on accelerating the development of animal husbandry mechanization opinion*, *Technical specification for innocent treatment of animal manure*, *the measures for the management of agricultural production development funds*, and *the livestock and poultry breeding waste recycling use job evaluation implementation plan*, etc. Regarding agricultural machinery, technical guidance, capital, and performance appraisal, the mechanization of animal husbandry and manure recycling were vigorously promoted, and the scale and intensification of animal husbandry production inhibited the carbon emissions from animal husbandry and promoted the high-quality development of animal husbandry [39]. On the other hand, the outbreak of H7N9 in 2017, African swine fever in 2018, and COVID-19 in 2019 hindered livestock breeding to a certain extent, thus inhibiting carbon emissions from livestock farming [40]. Therefore, although the total carbon emissions of the livestock industry in this stage increased in some years, the overall trend was a decreasing one year by year.

According to the line chart in Figure 2a, by region, carbon emissions from animal husbandry in eastern, central, and western China all peaked in 2006. From 2000 to 2017, the broken line trends of the eastern, western, and central regions were basically consistent. In terms of the specific carbon emissions of each region, the central region produced the largest carbon emissions, the western region was lower than the central region, and the eastern region produced the least. After 2017, the carbon emissions from animal husbandry in the eastern and central regions showed a significant downward trend, while the carbon emissions in the western region showed a significant upward trend, and the carbon emissions in the upper western region exceeded that in the central region. By type, the top three livestock animals contributing to the carbon emissions were non-cows (including scalper, buffalo, etc.), pigs, and sheep. After 2017, the carbon emissions from non-cows and sheep showed an upward trend, while the carbon emissions of pigs showed a downward trend. Possible reasons are that, for the eastern and central regions, in recent years, because of livestock breeding pollution, especially the serious problems in pig breeding, the central and local governments established a series of restrictive measures for the pig breeding industry in some economically developed southern provinces. Some southern provinces with pig breeding populations reduced carbon emissions to a certain extent to promote animal husbandry. For the western region, on the one hand, in order to stabilize the Chinese pork supply, the central and local governments vigorously supported the development of the pig industry and provided assistance in terms of capital, policy, and technology. On the other hand, in China, much of the high-quality grassland and pasture land are located in the western region, and the main source of animal husbandry carbon emissions is the large amounts of livestock such as sheep, buffalo, and especially non-cow. The annual number of average breeding cows grew yearly, increasing the trend of carbon emissions and contributing to the proportion of animal husbandry carbon emissions in the western region.

With the support of the ArcGIS10.2 (Environmental Systems Research Institute, Redlands, CA, USA) software, the data of 2000, 2010, and 2020 were selected, and the carbon emission level of the livestock industry in each province was divided into four grades: high region, slightly high region, moderate region, and low region using the natural discontinuous point method.

**Figure 3:**
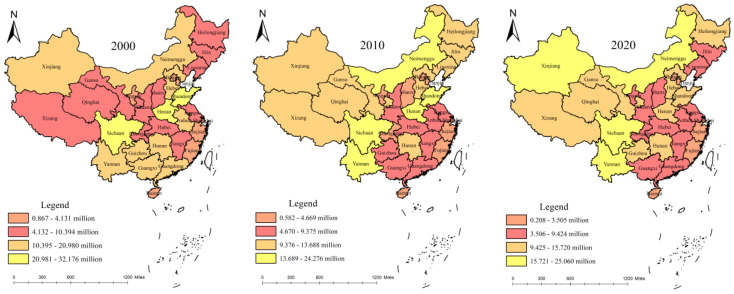
Spatial distribution pattern of carbon emissions from animal husbandry in 2000, 2010, and 2020.

Figure 3 shows that in 2000, the relative difference in the carbon emission levels from animal husbandry in China was obvious among the provinces, and there were many areas with moderate, slightly high, and high regions. The high regions were only in Sichuan, Henan, and Shandong provinces, whereas slightly high regions were in nine provinces: Xinjiang, Inner Mongolia, Hebei, Yunnan, Guangxi, Guizhou, Hunan, Anhui, and Guangdong. Compared to 2000, in 2010, the number of high, moderate, and low regions increased, the number of slightly high regions decreased, and the relative gap between the provinces narrowed. Among them, Inner Mongolia and Yunnan were upgraded from slightly high regions to high regions, while Guangdong, Guangxi, Guizhou, and other provinces retreated to a moderate region. At the same time, the preliminary highlights of China’s animal husbandry carbon cluster characteristics presented a concentrated zone “block” distribution. The slightly high regions such as Xinjiang, Tibet, Gansu, Inner Mongolia with the high regions such as Sichuan and Yunnan are connecting as an organic whole. The animal husbandry high and slightly high regions tended to be located in the western region. By 2020, the number of high regions decreased by two, but the number of slightly high regions increased again, and the relative gap among the provinces widened. Among them, Shandong and Henan provinces changed from high regions to slightly high regions, Xinjiang from a slightly high region to a high region, and Qinghai and Guizhou provinces from moderate regions to slightly high regions. At the same time, the distribution characteristics of the “block” of the concentrated contiguous area were further clarified. The regions with the high carbon emissions of the livestock industry and the regions with slightly high carbon emissions were obviously located in the western area, and the distribution characteristics of the “block” of the concentrated contiguous area were also further clarified, specifically, the carbon emissions and the regional distribution of the high and slightly high regions in Inner Mongolia, Xinjiang, Gansu, Qinghai, Tibet, Sichuan, and Yunnan provinces. The slightly high and moderate regional distributions are located in prairie areas such as Shandong, Henan, and Hebei provinces, and major grain-producing areas in three provinces in the northeast, and the low region is located in Zhejiang, Shanghai, Jiangsu, Fujian, and the southeast coastal provinces. In general, carbon emissions from animal husbandry showed a significantly decreasing spatial distribution pattern from the northwest to the southeast.

#### 3.1.2. Time Evolution Trend of Carbon Emission from Animal Husbandry

Previous studies on the dynamic evolution of carbon emissions from animal husbandry have mostly adopted the two-dimensional planar kernel density model. The limitation of this method is that only the two-dimensional kernel density curve of the research object can be selected to represent the year. In this paper, the three-dimensional kernel density curve of all the study years was drawn with the help of MATLAB software. It can very carefully, accurately, and completely reflect the dynamic evolution process of carbon emissions from the livestock industry in each province of China.

**Figure 4:**
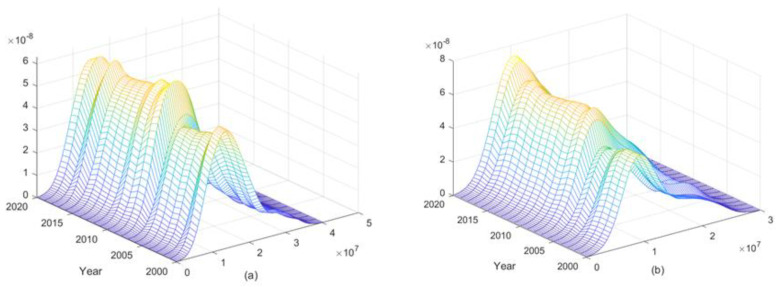

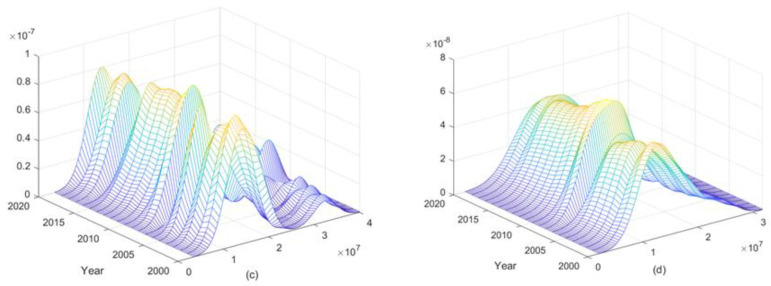
Dynamic evolution of carbon emissions from the livestock industry in China’s eastern, central, and western regions.

In order to clarify the overall distribution and change trends of carbon emissions from animal husbandry in different periods in the eastern, central, and western regions of China, we plotted the kernel density estimation of carbon emissions from animal husbandry for the whole country and the eastern, central, and western regions from 2000 to 2020, as shown in Figure 4.

Figure 4a depicts the distribution dynamics of carbon emissions from the livestock industry in China. From the movement of the wave peak, the main peak of the carbon emission distribution curve of the national livestock industry shifts to the right first and then to the left, indicating that carbon emissions from the national livestock industry experienced a change process of first increasing and then decreasing. The height of the main peak experiences a process of “upward-downward-rising”, and the width of the peak increases continuously, indicating that the relative gap among the carbon emissions from animal husbandry in various provinces in China narrowed first and then expanded.

There was a tailing phenomenon, but the scope of the right drag decreased, indicating that the difference between the provinces with the highest and lowest carbon emissions from animal husbandry is decreasing. Figure 4b–d, respectively, displays the features of three regional (eastern, central, and western) animal husbandry distribution dynamics of carbon emission levels. From the point of distribution location, the eastern part of the main nuclear density curve shows a trend of distribution to the left, along with the central region, and the western region shows a trend of right distribution, indicating that in recent years, animal husbandry carbon emissions in the eastern and central regions have declined. The eastern region had a faster downward trend than the central region, while the carbon emissions level of animal husbandry in the western region experienced an upward trend. The possible reason is that the eastern region has a more developed economy and a higher level of mechanization and technology in farming and animal husbandry, which promoted the carbon emission reduction goal of animal husbandry. However, the mechanization of agriculture and animal husbandry in western China is still in its initial stage, and the increases in production and income depend more on factor input than on technological progress. With the support of national policies to encourage the development of animal husbandry in western China, it is inevitable that carbon emissions will continue to increase in the short term. In terms of distribution extensibility, there was an obvious tailing phenomenon in the eastern and central regions, indicating a certain spatial difference between the provinces with the highest carbon emissions and other regions. The central region in particular formed a small wave to the right, and the main representation of most of the province formed a bigger difference. The reason may be that central Inner Mongolia’s autonomous region is a major animal husbandry province of the country; the natural resources are superior, it has much high-quality, grassland, pasture animal husbandry, rich resources, farming on a large scale, and the animal husbandry carbon level is higher. There is a certain spatial spillover effect on the carbon emissions from the livestock industry in the neighboring province, which affects or even increases this level and forms a higher level of carbon emissions area within a certain range. However, in the western region, there was no obvious tailing phenomenon, and the spatial distribution was balanced. The animal husbandry carbon emission levels in all provinces were basically the same, at a high level. The reason may be that China’s four big pastoral areas [32], in addition to the pastoral areas of Inner Mongolia, Xinjiang, Tibet, and Qinghai, are located in the western region. On the one hand, the highland pastoral areas have strong sunlight during the day, a large temperature difference between day and night, sunny days, and many natural advantages, such as areas for forage growth and sugar storage and adequate supplies of high-quality foraging. On the other hand, the high altitude and cold climate greatly reduce the possibility of epidemics and the outbreak of livestock infectious diseases. The survival rates of large livestock such as non-cow, sheep, and horses are high, and the average annual feeding volume is high. Therefore, the overall carbon emission level of the livestock industry in the region is high, and the difference is not obvious.

### 3.2. Spatial Correlation Analysis of Carbon Emission in the Livestock Industry

#### 3.2.1. Global Spatial Autocorrelation Analysis

**Figure 5:**
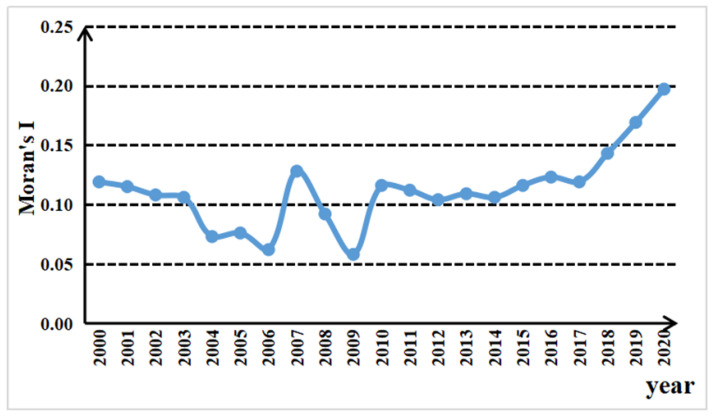
Global Moran index of carbon emissions from China’s livestock industry from 2000 to 2020.

As shown in Figure 5, the Moran index values of carbon emissions from China’s livestock industry were all greater than 0, indicating that there was a certain spatial correlation between carbon emissions from 2000 to 2020. Specifically, the change in the Global Moran index of carbon emissions from China’s livestock industry can be divided into three stages. The first stage (2000–2006) was the decline stage, and the Global Moran index decreased from 0.119 in 2000 to 0.062 in 2006, indicating that the spatial dependence of carbon emissions from China’s livestock industry was decreasing before 2006. The possible reason was the ministry of agriculture issuing “*the opinion on accelerating the development of animal husbandry*” to support and promote the development of animal husbandry and to reduce animal husbandry tax breaks, the animal slaughter tax, and related taxes. These measures aroused the enthusiasm of the country’s regional farmers and herders and promoted the development of animal husbandry, increasing carbon emissions. In the second stage (2006–2009), the Global Moran index rose first and then declined, with a large range and sharp fluctuation, reaching the peak and bottom point in 2007 and 2009, respectively. The possible reason is that, influenced by the world food crisis, the country had been to reducing the development of grain-consuming animal husbandry since 2004. However, the implementation of policies often has a time-lag effect, and the effect of policies was not realized until around 2007. After 2007, animal husbandry began to recover gradually, and the carbon emissions in each region changed from a decline to a slow growth. Thus, the spatial correlation of carbon emissions experienced a fluctuation stage of first rising and then declining. The third stage (2009–2020) was the rising stage. After experiencing the fluctuation period from 2006 to 2009, the Global Moran index began to rise yearly from 2009 to reach the peak of 0.197 in 2020. This indicated that over the past decade, with the close exchanges of economic activities in various regions, the technology and experience related to animal husbandry flowed more freely among regions, having a demonstrable effect on neighboring regions. As a result, the spatial dependence on carbon emissions increased annually, and the upward trend became more and more obvious. The spatial autocorrelation strengthened, and the spatial influence continues to exist.

#### 3.2.2. Local Spatial Autocorrelation Analysis

**Figure 6:**
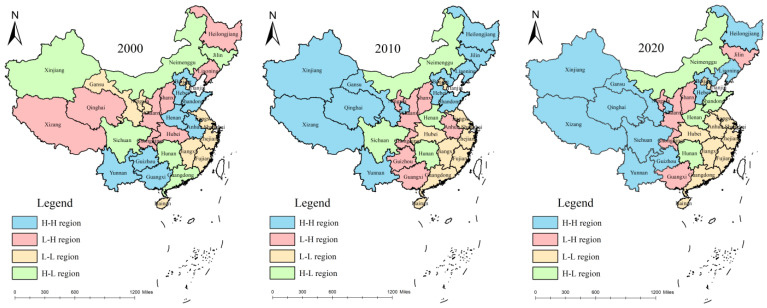
Spatial correlation of carbon emissions from animal husbandry in 2000, 2010, and 2020.

The scatter plots of the Local Moran index in 2000, 2010, and 2020 were calculated and drawn with the help of Stata15.1 software. According to the four quadrants of the scatter plot, the spatial differences in carbon emissions from animal husbandry in each province can be divided into four categories: the first quadrant is the high (H–H) aggregation area, indicating that the carbon emissions in this region and adjacent regions were high, and the spatial correlation was with the area with a high emission level. The second quadrant is the low–high (L–H) aggregation area, which indicates that the carbon emission level of the livestock industry in this region was low while that in adjacent regions was high, and the spatial correlation shows that the emission level of the livestock industry was medium. The third quadrant is the low–low (L–L) aggregation area, which means that the carbon emissions from animal husbandry in this area and adjacent areas were low, and the spatial correlation is with the area with a low emission level. The fourth quadrant is the high and low (H–L) cluster area, indicating that the carbon emission level in this region was higher than that in adjacent regions, and the spatial correlation is manifested as a spillover effect. The Local Moran scatter plots for 2000, 2010, and 2020 can be used to plot the spatial correlation maps for the corresponding years (Figure 6).

Further analysis showed that in 2000, the L–L concentration areas of carbon emissions from animal husbandry were mainly distributed in Gansu and Ningxia in the western region and Jiangsu, Shanghai, Zhejiang, Fujian, and Jiangxi in the eastern region, accounting for about 10.47% of the study area. H–H clusters were mainly distributed in Yunnan, Guizhou, and Guangxi in southwest China and Hebei, Shandong, Henan, and Anhui in east China, accounting for 15.18% of the study area. In 2010, regarding the animal husbandry carbon type, great changes occurred. H–H accumulation increased from 15.18% to 58.12%, although four provinces, Guangxi, Guizhou, Henan, and Anhui, no longer belonged to the group. The newly added Heilongjiang, Liaoning, and Jilin provinces, three major grain-producing areas, and the three prairie pastoral areas of Xinjiang, Qinghai, and Tibet provinces, also received a promotion. The area of L–L agglomeration decreased slightly from 10.47% to 8.99%. In 2020, the area of the H–H cluster continued to expand, and after Sichuan and Guizhou provinces were added, it almost covered the whole western region, accounting for 61.43%. The area of L–L agglomeration increased slightly, and the proportion of Anhui Province in the L–L agglomeration increased to 10.44%, which was almost the same as that in 2000.

In general, from 2000 to 2020, the H–H aggregation phenomenon of carbon emissions from animal husbandry in China continuously enhanced, expanding from the initial southwest corner to almost the entire western region, and three of the four pastoral areas were included, becoming the largest aggregation type. However, the L–L accumulation area showed little change, mainly distributed in the eastern coastal areas, covering the Yangtze River Delta and Pearl River Delta. The possible reason is that the H–H aggregation phenomenon in the western region was closely related to the national Western development strategy. It promoted the rapid development of animal husbandry with advantaged foraging resources, climate characteristics conducive to the growth of herbage, and a long history of nomadic tradition. As a result, a high-level carbon emission area was formed, with Inner Mongolia, Xinjiang, Qinghai, and Tibet as the core. The phenomenon of H–H aggregation in northeast China occurred because the region relies on the advantages of the northeast plain as the main grain-producing area, forming the animal husbandry emission zone in the farming area, supported by byproducts such as grain production and livestock feed processing. In the L–L cluster located in the southeast coastal area, because of its high level of economic development and developed industry, people’s demand for animal husbandry products can be met by importing from other provinces, so animal husbandry is a relatively low proportion in agriculture and produces less carbon emissions. At the same time, the high mechanization and technology of agriculture have strongly promoted the carbon emission reduction goal of animal husbandry, explaining the spatial distribution phenomenon of L–L aggregation.

### 3.3. Analysis of Influencing Factors of Carbon Emission in Animal Husbandry

This paper selected the random effects panel *Tobit* model to estimate measurement, on the one hand; compared with the fixed effect panel *Tobit* model, the random effects panel *Tobit* consistency model can be estimated [41]. On the other hand, this can effectively avoid the results of the biased OLS estimate [42].

Using the Stata15.1 software (Stata-Corp LLC, College Station, TX, USA) to calculate the random effects panel *Tobit* model for maximum likelihood estimation, the regression results are shown in Table 8:

As a result of the four samples, the LR inspection results were strongly rejected. “$H_{0}:\sigma_{\mu }=0$”(sigma_μ=0) means that the individual random effects model should not be mixed regression; at the same time, the rho values in the four samples, 0.829, 0.884, 0.898, and 0.815, were greater than 0.5, proving that the selected random effects panel *Tobit* model was more reasonable (Table 9) [43].

#### 3.3.1. National Outcome Analysis

Regarding the positive regression coefficient of the industrial structure of animal husbandry carbon emissions, the 1% level of significance test indicated that the industrial structure of animal husbandry carbon played a stronger role in promotion. The possible reason is that, as Chinese living standards are increasing day by day, the demands for livestock products such as meat, milk, and eggs are also growing. Therefore, the status of animal husbandry in China’s agricultural industrial structure has been constantly improved, which makes the industrial structure factor an important factor affecting the carbon emissions from China’s animal husbandry in the future. Therefore, adjusting the industrial structure may be an effective way to achieve carbon emission reduction in the livestock industry. The regression coefficient of the urbanization level for carbon emissions from animal husbandry was negative and passed the significance test at the level of 1%, indicating that the increase in the urbanization rate had a strong inhibitory effect on carbon emissions from animal husbandry, which was also consistent with the research conclusion of Yao [9]. The possible reasons are as follows: In recent years, China’s urbanization rate has been increasing annually, the transfer of rural labor has intensified, and non-agricultural income has increased. The negative regression coefficient of the agricultural mechanization level of animal husbandry carbon, through the 10% level of the significance test, indicated that the effect of improvement of agricultural mechanization on livestock emissions was inhibiting, possibly because, on the one hand, with the constant improvement of the agricultural mechanization level, required by the traditional agriculture of cattle and buffalo, the number of newborn pigs and other animals is declining. Old equipment has been gradually replaced by new agricultural machinery and equipment on a large scale. On the other hand, the improvement of the agricultural mechanization level has promoted the transformation of traditional free-range animal husbandry to a large-scale and intensive production mode. Through the establishment of a series of biogas projects, livestock manure has been transformed into energy through professional treatment and utilization, significantly reducing the carbon emissions from animal husbandry. The positive regression coefficient of carbon production efficiency in animal husbandry passed the 1% level of the significance test, but the coefficient estimate was only 0.0036156, suggesting that livestock production efficiency to some extent promoted the growth of animal husbandry carbon emissions. However, compared with other factors, the promoting effect was not obvious. The possible reason is that, in recent years, the carbon emissions of the livestock industry in China have shown a downward trend and a slow decline, while the total output of the livestock industry has shown an upward trend and a slow increase. The change range of carbon production efficiency of the livestock industry is small, so the promotion effect on carbon emissions of the livestock industry has been limited. The regression coefficient of the total population was positive and passed the significance test at the level of 1%, which indicated that the increase in the total population had a strong promotional effect on the carbon emissions from the livestock industry. The possible reason is that the increase in the total population led to the rapid growth in demand for animal husbandry products, which in turn promoted the rapid development of animal husbandry. With the continuous adjustment of the national fertility policy, especially since the national three-child policy was begun on 31 May 2021, the growth rate of China’s population may accelerate in the future, so the promoting effect of the total population factor on the growth of carbon emissions from animal husbandry will continue to strengthen. The regression coefficient of farmers’ income levels was positive and passed under the 1% level of the significance test, indicating that the improvement in farmers’ income levels promoted an increase in carbon emissions. There may be two aspects to analyze from the farmers and the government. On the one hand, from the farmers’ perspectives, the economic benefits of livestock breeding are far higher than those of the planting industry. In order to improve the level of personal income, the majority of farmers will inevitably choose to expand the channels of personal income by engaging in animal husbandry. On the other hand, from the government’s perspective, no matter the previous poverty alleviation or the current rural revitalization strategy, the government will take the development of animal husbandry as an important channel to improve farmers’ incomes and use a variety of policies and means to support and encourage farmers to achieve poverty alleviation through the development of animal husbandry. At the same time, the improvement in the farmers’ income levels also promotes an increase in farmers’ demand for meat, milk, eggs, and other animal husbandry products, which further promotes the growth of carbon emissions from animal husbandry.

#### 3.3.2. Analysis of Results by Region

Based on the natural geographical environment and economic and administrative divisions, this paper divided the country into three major regions: eastern, central, and western. It can be seen from Table 8 that the change in industrial structure factors was the most important element leading to the increase in carbon emissions from animal husbandry in the three regions. The industrial structures of the three major areas of animal husbandry carbon regression coefficients were all positive, and all passed the 1% level of the significance test, indicating that the industrial structure of national regional animal husbandry carbon emissions had a stronger positive role in promoting emissions. The reason may be the increase in China’s demand for meat. The status of animal husbandry in China’s agricultural industrial structure will continue to improve, which maintains the industrial structure as an important factor affecting the carbon emissions from China’s animal husbandry in the short term. The regression coefficient of agricultural economic development level in the east passed only the 1% level of the significance test, while in the Midwest, it did not pass the significance test. The possible reason is that in the more developed eastern region, people’s living standard is higher, so the demand for high-quality agricultural products, especially animal husbandry products, is bigger, which results in higher animal husbandry carbon emissions. The regression coefficients of urbanization level features in the three major areas were negative, and the coefficients were bigger, indicating that improving the urbanization rate is crucial to inhibiting animal husbandry carbon emissions, but the regression coefficient of the urbanization level in the eastern region of the country only passed the 1% level of the significance test; the central and western regions did not pass the test of significance. A possible reason is that the eastern region is densely populated, and traffic is convenient. With a “take the lead in” eastern regional strategic support, the urbanization rate increased rapidly. The central and western regions were part of the “central rise” and “western development” and other regional strategic support but were limited by the natural geographical environment constraints, such as sparse population, traffic inconvenience, brain drain, and the slow process of urbanization. Although in recent years, the region has been promoted, overall, it is still far behind the eastern region. Therefore, the impact on carbon emissions from animal husbandry has been limited. The regression coefficient of agricultural mechanization level in the eastern region was positive and passed the 5% level of the significance test; the central region was negative and passed the 1% level of the significance test; the western region did not pass the test of significance. A possible reason is that the eastern region’s economic development level was higher, its agricultural mechanization was developed to a higher level, and enhanced space was limited. Therefore, in promoting animal husbandry carbon emissions, the economic development level was lower than in the eastern and central regions, the agricultural mechanization level expansion space was larger, and its contribution to the animal husbandry carbon reduction potential was tremendous. Therefore, it had a significant inhibitory effect on the carbon emissions from animal husbandry. Western China is limited by its level of economic development compared with the eastern region regarding the low level of mechanization. Its influence on animal husbandry carbon emissions was not obvious, so there were no significant regression results. The regression coefficient of animal husbandry carbon production efficiency in the east, central, and western regions were all positive and passed the 1% level of the significance test but had smaller values of the regression coefficient. The animal husbandry production efficiency played a certain role in promoting animal husbandry carbon emissions, but the promoting effect was not obvious, which was also consistent with the results from the entire sample. The regression coefficient of the total population was positive in the eastern and western regions and passed the significance test at the level of 1%. The western region is mostly inhabited by ethnic minorities, with low levels of urbanization and industrialization, and the increased population mostly depends on the development of agriculture and animal husbandry. The regression coefficient of dietary structure in the eastern region was negative, and in the western region, it was positive by 5% and 10% levels of significance, respectively. The possible reason is that as the living standard rose in the east, green policies, environmental protection, and health consciousness constantly increased, reducing the consumption of meat products and leading to animal husbandry carbon inhibition. On the contrary, in the western region, residents consumed more meat products after improving their living standards, thus promoting the increase in carbon emissions from animal husbandry. The regression coefficient of farmers’ income levels was positive in the eastern and western regions, but only the eastern and central regions passed the significance test at the 10% and 1% levels, respectively, and the coefficient estimate of the eastern region was smaller than that of the central region. The possible reason is that the eastern region is economically developed, and the farmers’ income levels are high; therefore, improving the farmers’ income level is mostly achieved through technological progress and efficiency improvement, while the central and western regions are relatively backward economically. Therefore, the most effective means to improve the farmers’ income level is to increase the factor input. Therefore, the improvement of the farmers’ income level in the central region had a stronger promoting effect on the carbon emissions from animal husbandry than that in the eastern region.

## 4. Conclusions and Suggestions

### 4.1. Research Conclusions

We considered temperature variation factors of the discharge coefficient method to calculate China’s 31 provinces’ (municipalities directly under the central government, autonomous regions) livestock emissions for 2000–2020, using the kernel density estimation model, spatial autocorrelation, and the Tobit model for the carbon emissions, spatial distribution, and time evolution of animal husbandry, analyzing the factors influencing the research conclusion as follows:

First, from 2000 to 2020, the carbon emission level of the livestock industry in China experienced four stages, namely, rapid rise, rapid decline, slow rise, and fluctuation decline, showing a downward trend on the whole. By region, carbon emissions in the eastern and central regions showed a downward trend, while carbon emissions in the western regions showed an upward trend. By type, the top three livestock animals contributing to the carbon emissions from animal husbandry were non-cows (including scalpers, buffalo, etc.), pigs, and sheep. Since 2017, the carbon emissions from non-cows and sheep have been on the rise, while the carbon emissions from pigs have been on the decline.

Second, in terms of time, the relative gaps in carbon emissions among the provinces first narrowed and then widened, and the difference between the provinces with the highest and lowest carbon emissions from animal husbandry continued to narrow. The spatial agglomeration of carbon emissions from animal husbandry in China has been increasing, gradually forming the spatial characteristics of “high agglomeration and low agglomeration”. The regions with high carbon emissions from animal husbandry and the regions with slightly high carbon emissions were distributed in Inner Mongolia, Xinjiang, Gansu, Qinghai, Tibet, Sichuan, Yunnan, and other grassland and pastoral provinces. The slightly high and moderate regions were distributed in Shandong, Henan, Hebei, and northeast grain-producing provinces, while the low regions were distributed in the southeast coastal provinces, such as Zhejiang, Shanghai, Jiangsu, and Fujian. The overall spatial distribution pattern of carbon emission from livestock husbandry decreased significantly from the northwest to the southeast.

Third, across the nation, industrial structure, population, and farmers’ income levels had a strong positive promoting effect on animal husbandry carbon emissions, and the urbanization level and agricultural mechanization level of animal husbandry had a significant inhibitory effect on carbon emissions. The dietary structure and agricultural economic development level had a promoting but not significant effect on animal husbandry carbon. From the perspective of the eastern region, the industrial structure, agricultural economic development level, total population, farmers’ income level, agricultural mechanization level, and other factors played a strong positive role in promoting carbon emissions from animal husbandry, while the urbanization level and dietary structure had a significant effect on carbon emissions from animal husbandry. From the perspective of the central region, industrial structure and farmers’ income levels played a strong positive role in promoting carbon emissions from animal husbandry, while the level of agricultural mechanization had a significant inhibitory effect on carbon emissions from animal husbandry. From the perspective of western China, industrial structure, total population, and dietary structure had strong positive promoting effects on carbon emissions from animal husbandry, while the urbanization level had a negative but insignificant effect on carbon emissions from animal husbandry.

### 4.2. Policy Proposal

These findings have important policy implications for national and regional carbon emission reduction in the livestock industry.

First, we should pay attention to the basic spatial pattern of carbon emissions from animal husbandry, as determined by natural resource endowment and location conditions. For some time to come, the western region, especially in prairie area provinces, will be the core of the animal husbandry carbon emissions growth area. Animal husbandry should be focused on carbon emission reduction in the western region by following the rules of scale, science, and clean farming; adjusting the feed structure; breeding new varieties; and improving waste disposal methods, to carry out actions to reduce emissions.

Second, it should be recognized that the industrial structure is an important factor of carbon emissions in China’s livestock industry. The government at all levels must try to gradually expand taxation on the basis of a carbon tax, promote animal husbandry industrial structure upgrades, and create a scientific plan for the future structure of livestock breeding of animals high on the greenhouse gas emissions livestock farming scale (such as non-cow, sheep, and pigs) with reasonable control, so as to speed up the provincial animal husbandry carbon neutral goals and ensure high-quality animal husbandry development.

Third, different strategies should be adopted according to the different conditions in the eastern, central, and western regions to achieve carbon emission reduction targets in animal husbandry. For the eastern region, it is necessary to accelerate the people-oriented new urbanization and further reduce the dependence of farmers’ income increase on the development of animal husbandry. For the central region, we should vigorously promote the development of the agricultural mechanization level, strengthen the guidance and inclination of the agricultural machinery subsidy policy, encourage farmers to buy high-efficiency and energy-saving new agricultural machinery, and improve the level of agricultural mechanization. For the western region, the relevant spirit of the *Program for the Development of Food and Nutrition in China (2014–2020)* should be thoroughly implemented, and residents should be encouraged to develop green eating habits with plant food as the main source and animal food as the supplement, so as to avoid the phenomenon of a large increase in the consumption of animal husbandry products with the rise in income.

### 4.3. Discussion

This paper has the following shortcomings: First, considering the availability and operability of data, 31 provincial–level administrative units in China were selected as the research objects. If more micro and specific regions (prefecture-level cities and county-level cities) are selected in the future, the spatial-temporal characteristics of carbon emissions from animal husbandry will be more accurately reflected. Second, because of the different physical and geographical conditions, there were significant regional differences. In the future, specific zoning research can be conducted in combination with the different development goals of each region. Third, although livestock intestinal fermentation and waste management are discussed in this paper, for an accurate calculation of carbon emissions, other processes must be considered, such as feed grain planting, transportation, raising, and product processing. In the future, if we can acquire the relevant data and use the method of the whole life-cycle method of carbon emissions in the overall process of animal husbandry, the results will be more accurate and reliable.

## Figures and Tables

**Table 1:** Emissions coefficient of $CH_{4}$ of intestinal fermentation $\left({\mathrm{kg}}^{-1}\cdot {\mathrm{head}}^{-1}\right)$.

Species	Cow	Non-Cow	Donkey	Camel	Horse	Goat	Sheep	Buffalo	Pig
coefficient	61.00	47.00	10.00	46.00	18.00	5.00	5.00	55.00	1.00

**Table 2:** Emissions coefficient of $CH_{4}\;$ of waste management $\left({\mathrm{kg}}^{-1}\cdot {\mathrm{head}}^{-1}\right)$.

Species	Cold (<15)	Warm (15~25)	Hot (>25)
sheep	0.10	0.15	0.20
goat	0.11	0.17	0.22
camel	1.28	1.92	2.56
horse	1.09	1.64	2.19
donkey	0.60	0.90	1.20
poultry	0.01	0.02	0.02
non-cow	1.00	1.00	1.00
buffalo	1.00	2.00	2.00

**Table 3:** Emissions coefficient of $CH_{4}\;$ of waste management of cow $\left({\mathrm{kg}}^{-1}\cdot {\mathrm{head}}^{-1}\right)$.

**Temperature**	**≤10**	**11**	**12**	**13**	**14**	**15**	**16**	**17**	**18**
coefficient	9	10	10	11	12	13	14	15	16
**Temperature**	**19**	**20**	**21**	**22**	**23**	**24**	**25**	**26**	**≥27**
coefficient	17	18	20	21	23	24	26	28	31

**Table 4:** Emissions coefficient of $CH_{4}\;$ of waste management of pig $\left({\mathrm{kg}}^{-1}\cdot {\mathrm{head}}^{-1}\right)$.

Temperature	<10	10~14	15~18	19~21	22~24	25~26	27	≥28
coefficient	2	2	3	4	5	6	7	7

**Table 5:** Emissions coefficient of $N_{2}O\;$ of waste management $\left({\mathrm{kg}}^{-1}\cdot {\mathrm{head}}^{-1}\right)$.

Species	Cow	Non-Cow	Donkey	Camel	Horse	Goat	Sheep	Buffalo	Pig	Poultry
coefficient	1.00	1.39	1.39	1.39	1.39	0.33	0.33	1.34	0.53	0.02

**Table 6:** Influencing factors of carbon emission of animal husbandry.

Influence Factor	Variable Meaning and Unit
industrial structure	Gross output of regional animal husbandry/Gross output of regional AFAF (%) ^1^
urbanization level	Regional permanent urban population/Total regional population (%)
level of agricultural economic development	Gross output of regional AFAF/The number of agricultural labor force in the region (per capita)
agricultural mechanization level	Total power of regional agricultural machinery/Regional crop sown area (KW/Ha)
carbon production efficiency of animal husbandry	Carbon emissions from regional animal husbandry/Gross output of regional animal husbandry (Ton/Ten thousand yuan)
population gross	The sum of permanent urban and rural population
dietary pattern	Per capita consumption of meat, milk, and eggs in rural areas (kg)
farmer income level	Per capita disposable income of rural households (yuan)

^1^ AFAF stands for agriculture, forestry, animal husbandry, and fishery.

**Table 7:** Expected results of variables.

Influence Factor	Expected Relationship of Variables
industrial structure [32]	+
urbanization level [33]	−
level of agricultural economic development [34]	+
agricultural mechanization level [34]	−
carbon production efficiency of animal husbandry [32]	−
population gross [33]	+
dietary pattern	+
farmer income level [34]	+

Note: “+” represents the positive promotion, “−” represents the negative inhibition.

**Table 8:** Regression results of Tobit model.

	All		Eastern		Central		Western	
variable	coefficient	*p*-value	coefficient	*p*-value	coefficient	*p*-value	coefficient	*p*-value
structure	2057.09	0.000 ***	923.5562	0.005 ***	7655.906	0.000 ***	1889.975	0.000 ***
economy	0.0005146	0.658	0.0028379	0.002 ***	0.0015105	0.608	0.0059441	0.130
urban	−1073.124	0.000 ***	−434.372	0.008 ***	−489.9366	0.184	−502.3993	0.123
machine	−3.894607	0.083 *	21.72374	0.023 **	−10.73045	0.002 ***	1.488717	0.546
efficiency	0.0036156	0.000 ***	0.0227908	0.000 ***	0.0260597	0.000 ***	0.0036115	0.000 ***
ln population	307.9129	0.000 ***	202.973	0.002 ***	177.0255	0.105	715.3062	0.000 ***
ln food	15.88383	0.776	−170.0215	0.026 **	149.3371	0.108	141.2574	0.061 *
ln income	133.1183	0.001 ***	102.912	0.066 *	305.8225	0.001 ***	43.85069	0.503
cons	−5800.22	0.000 ***	−1907.646	0.002 ***	−6476.991	0.000 ***	−5961.439	0.000 ***

Note: ***, **, and * indicate significance at 1%, 5%, and 10% levels, respectively.

**Table 9:** *LR* test result.

	All	Eastern	Central	Western
*sigma_μ*	491.0035	416.4851	620.2643	381.7481
*sigma_e*	222.9122	150.5841	209.5508	182.1469
*rho*	0.8291119	0.8843882	0.8975559	0.8145567

## Data Availability

Data are available on request due to restrictions, e.g., privacy or ethical. The data presented in this study are available on request from the corresponding author. The data are not publicly available due to the strict management of various data and technical resources within the research teams.

## Appendix A

**Table A1:** Annual mean temperature of representative years from 2000 to 2020.

City	2000 Year	2004 Year	2008 Year	2012 Year	2016 Year	2020 Year
Beijing	12.8	13.5	13.4	12.9	13.8	13.8
Tianjin	12.9	13.2	13.3	12.5	13.8	13.8
Shijiazhuang	13.9	14.3	14.6	14.0	14.6	14.7
Taiyuan	10.7	10.9	10.9	10.7	11.2	11.2
Hohhot	7.5	8.0	7.4	7.2	7.1	7.0
Shenyang	8.3	9.6	8.6	7.4	8.8	9.2
Changchun	5.6	7.1	7.2	5.2	6.6	7.1
Harbin	4.6	5.8	6.6	4.6	5.0	5.4
Shanghai	17.2	17.5	17.2	16.9	17.6	17.8
Nanjing	16.4	16.9	16.1	16.0	16.8	17.1
Hangzhou	17.2	17.8	17.5	17.1	18.2	18.3
Hefei	16.7	16.6	16.4	16.5	17.0	16.2
Fuzhou	20.5	20.8	20.4	20.2	21.0	21.5
Nanchang	17.9	18.8	18.5	18.0	19.0	19.1
Jinan	14.5	14.8	14.6	14.3	15.4	15.0
Zhengzhou	15.0	15.5	15.6	15.5	16.4	16.5
Wuhan	17.7	18.3	17.6	16.4	17.3	17.1
Changsha	17.1	18.3	18.3	17.6	17.5	17.5
Guangzhou	22.5	22.8	22.4	21.7	21.9	22.7
Nanning	21.5	21.5	20.8	21.4	22.3	22.1
Haikou	24.5	24.7	23.4	24.6	24.6	25.3
Chongqing	18.2	18.4	18.5	18.3	19.5	19.2
Chengdu	16.6	16.2	16.3	15.9	16.8	16.6
Guiyang	13.8	14.6	14.1	13.7	15.3	14.9
Kunming	15.6	15.6	15.4	16.3	15.8	16.5
Lhasa	8.4	8.6	8.9	9.6	9.5	9.7
Xian	14.5	15.4	14.9	14.2	15.8	15.2
Lanzhou	11.0	10.9	10.6	7.5	8.2	8.1
Xining	5.8	5.8	5.7	5.2	6.6	6.1
Yinchuan	9.6	10.3	9.9	9.8	10.7	10.7
Urumqi	7.3	8.0	8.7	7.4	8.4	8.7

### Description of the Data on Cows

Among them, the statistics of cattle feeding quantities need to be calculated in sections because of the different years and different statistical indicators. For the cows, the statistical data time limit of cows are 2000–2020. For the non-cows, the 2000–2007 statistical yearbook was used to distinguish the scalper and buffalo; therefore, the carbon emissions of scalpers and buffaloes were calculated according to different emission coefficients. From 2007–2020, due to the statistical yearbook collectively referring to non-cows and buffalo as the non-cows; therefore, only to use the non-cows emission coefficients calculating. The specific data are shown in cow.xlsx.
